# The Use of Pre-Chemoradiotherapy Total Masseter Muscle Volume as a Novel Predictor of Radiation-Induced Trismus in Locally Advanced Nasopharyngeal Carcinoma Patients

**DOI:** 10.3390/tomography10010007

**Published:** 2024-01-10

**Authors:** Efsun Somay, Erkan Topkan, Umur Anil Pehlivan, Busra Yilmaz, Ali Ayberk Besen, Huseyin Mertsoylu, Berrin Pehlivan, Ugur Selek

**Affiliations:** 1 Department of Oral and Maxillofacial Surgery, Faculty of Dentistry, Baskent University, Ankara 06490, Turkey; efsuner@gmail.com; 2 Department of Oral and Maxillofacial Surgery, Faculty of Dentistry, University of Kyrenia, Kyrenia 9265, Cyprus; 3 Department of Radiation Oncology, Faculty of Medicine, Baskent University, Adana 01120, Turkey; 4 Department of Radiology, Faculty of Medicine, Baskent University, Adana 01120, Turkey; uapehlivan@gmail.com; 5 Department of Oral and Maxillofacial Radiology, School of Dental Medicine, Bahcesehir University, Istanbul 34353, Turkey; busra.yilmaz1@bau.edu.tr; 6 Clinics of Medical Oncology, Adana Seyhan Medical Park Hospital, Adana 01120, Turkey; besenay@gmail.com; 7 Clinics of Medical Oncology, Istinye University, Adana Medical Park Hospital, Adana 01120, Turkey; mertsoylu@hotmail.com; 8 Department of Radiation Oncology, School of Medicine, Bahcesehir University, Istanbul 34450, Turkey; berrinpehlivan@gmail.com; 9 Department of Radiation Oncology, School of Medicine, Koc University, Istanbul 34450, Turkey; ugurselek@yahoo.com

**Keywords:** masseter muscle volume, concurrent chemoradiotherapy, nasopharyngeal cancer, radiation-induced trismus

## Abstract

Background: We sought to determine whether pretreatment total masseter muscle volume (TMMV) measures can predict radiation-induced trismus (RIT) in patients with locally advanced nasopharyngeal carcinoma (LA-NPC) receiving concurrent chemoradiotherapy (C-CRT). Methods: We retrospectively reviewed the medical records of LA-NPC patients who received C-CRT and had pretreatment maximum mouth openings (MMO) greater than 35 mm. MMO of 35 mm or less after C-CRT were considered RIT. We employed receiver operating characteristic (ROC) curve analysis to explore the correlation between pre-treatment TMMV readings and RIT status. Results: Out of the 112 eligible patients, 22.0% of them received a diagnosis of RIT after C-CRT. The optimal TMMV cutoff that was significantly linked to post-C-CRT RIT rates was determined to be 35.0 cc [area under the curve: 79.5%; sensitivity: 75.0%; and specificity: 78.6%; Youden index: 0.536] in the ROC curve analysis. The incidence of RIT was significantly higher in patients with TMMV ≤ 5.0 cc than in those with TMMV > 35.0 cc [51.2% vs. 8.7%; Odds ratio: 6.79; *p* < 0.001]. A multivariate logistic regression analysis revealed that pre-C-CRT MMO ≤ 41.6 mm (*p* = 0.001), mean masticatory apparatus dose V56.5 ≥ 34% group (*p* = 0.002), and TMMV ≤ 35 cc were the independent predictors of significantly elevated rates of RIT. Conclusion: The presence of a smaller pretreatment TMMV is a reliable and independent novel biological marker that can confidently predict higher RIT rates in LA-NPC patients who receive C-CRT.

## 1. Introduction

Nasopharyngeal carcinoma (NPC), a malignant tumor arising from the epithelial cells of the nasopharynx, is typically managed using radiotherapy (RT) for early-stage disease and concurrent chemoradiotherapy (C-CRT) with intensity-modulated radiotherapy (IMRT) for locally advanced-stage disease [1,2,3]. However, the benefits of C-CRT, such as improved locoregional control and survival rates, often come at the cost of severe late side effects that can negatively impact patients’ quality of life [4]. Radiation-induced trismus (RIT) is a notable late side effect of RT or C-CRT, with an incidence ranging from 28% to 42% [5]. However, it is often less respected despite its substantial adverse consequences on the patient’s functional abilities and quality of life metrics.

Trismus, also known as lockjaw, is a condition that can be attributed to several factors, including but not limited to temporomandibular joint (TMJ) disorders, tumors, surgery, RT/C-CRT to the head and neck, and TMJ interventions for managing infections and connective tissue disorders [6]. The formation of RIT is attributed to RT-induced inflammation and fibrosis occurring in the intramuscular region and associated raphes of the masseter muscle, namely the strongest masticatory muscle [7,8]. Masha et al. [7] established that applying a maximum dose of RT > 44 Gy to the masseter muscle region was associated with RIT in a cohort of 35 patients with head and neck tumors (*p* = 0.003). In addition, recent studies have confirmed that the incidence rates of RIT may be affected by hypoxia and inflammation markers, such as pre-C-CRT hemoglobin-to-platelet ratios (HPR), neutrophil-to-lymphocyte ratios (NLR), and hemoglobin values [9,10,11].

Cancer cachexia is characterized by muscle wasting (sarcopenia) and weight loss, often accompanied by a reduction in fat mass. Systemic inflammation and cytokine storms are the main contributing factors to this clinical condition [12,13]. The evaluation of sarcopenia in head and neck cancer (HNC) through imaging-based studies includes the utilization of the skeletal muscle index. This index is determined by dividing the cross-sectional area of skeletal muscle at either the C3 or L3 level by the square of the individual’s height in meters [12,14]. Scholarly discourse has recently focused on the masseter muscle, an essential component of the masticatory apparatus, as an alternative indicator for evaluating sarcopenia. A reduction in the volume of the masseter muscle can result in decreased mastication capability, ultimately resulting in malnutrition [15]. Disusing the masseter muscle and other muscles involved in the mastication process may cause muscular atrophy and loss of volume, resulting in different degrees of trismus or heightened vulnerability to developing RIT.

A recently published article has reported a direct correlation between the pretreatment masseter muscle volume and the survival outcomes of patients with locally advanced NPC (LA-NPC) who received definitive C-CRT [16]. It is currently unknown whether the pre-C-CRT volume of the masseter muscle affects RIT rates, despite its known connection to disease prognosis and cachexia. Hence, our current research aimed to investigate the potential impact of pre-treatment total masseter muscle volume (TMMV) on the RIT rates in LA-NPC patients who have undergone C-CRT.

## 2. Patients and Methods

### 2.1. Ethical Approval

The retrospective study protocol adhered to the official guidelines and amendments of the Declaration of Helsinki with utmost accuracy. Baskent University Faculty of Medicine’s Ethics and Scientific Committee granted approval before the collection of patient data, ensuring complete adherence to ethical standards (project No: KA23/196). 

### 2.2. Study Population

A retrospective analysis was conducted on patients diagnosed with LA-NPC who received C-CRT and were evaluated at the Departments of Radiation Oncology, Radiology, and Dentistry at Baskent University Faculty of Medicine between January 2010 and January 2023. Patients were required to be at least 18 years old, have been diagnosed with nasopharyngeal squamous cell cancer through histopathological analysis, undergone a neck MRI evaluation before treatment, have been classified as LA-NPC (T1-2N1-3M0 or T3-4N0-3M0) according to the eighth edition of the AJCC staging framework, and did not have a diagnosis of temporomandibular disorder (TMD) or trismus before C-CRT, based on the current diagnostic criteria for TMD (Diagnostic criteria (DC)/TMD), to be eligible for the study [17,18]. 

The exclusion criteria included immunosuppressive drug usage within 30 days before C-CRT, blood transfusions within 90 days, evidence of dehydration, evidence of acute and chronic infectious diseases, being treated for a local recurrence, a prior history of surgery and/or RT to the head and neck region, and a follow-up duration of less than 6 months. Patients who had missing central incisors in their upper or lower jaws, had previously undergone surgery for TMJ issues, TMJ ankylosis, head or neck trauma, muscle-related pain, or myofascial pain syndrome, and those with primary tumors or lymph node invasion in their masticatory muscles were also excluded from the study.

### 2.3. Treatment Protocol

Our institutional gold standard treatment approach for LA-NPC is simultaneous integrated boost intensity-modulated radiotherapy (SIB-IMRT). The target volumes for RT were determined by using pretreatment co-registered computed tomography (CT), 18-fluorodeoxyglucose-positron emission tomography (PET)-CT, and/or magnetic resonance imaging (MRI) scans of both the involved nasopharyngeal primary and the whole neck. The target volumes and corresponding RT doses were as previously described [19]. To provide a brief overview, the doses administered to the planning target volumes (PTVs) were as follows: 70.0 Gy for high-risk-, 59.5 Gy for intermediate-risk-, and 54.0 Gy for low-risk PTVs, delivered using single daily fractions over a period of 33 days [19]. The treatment plan mandated the administration of 1 to 3 cycles of concurrent chemotherapy, comprising cisplatin and 5-fluorouracil. All patients were recommended to undergo adjuvant treatment, which involved two cycles of the same chemotherapy regimen. Necessary supportive care measures, such as analgesic and antiemetic medications, were administered whenever required.

### 2.4. Baseline Total Masseter Muscle Volume Measurements

The masseter muscle is a quadrangular muscle that consists of two divisions: a superficial division and a deep division. Its superficial division originates from a thick aponeurosis located on the temporal process of the zygomatic bone and the anterior two-thirds of the inferior border of the zygomatic arch. The superficial division fibers attach to the lower part of the mandibular ramus and the mandibular angle (masseteric tubercle) by going downwards and backward over the deep portion. The whole zygomatic arch serves as the origin for the deep division of the masseter muscle. The fibers originate superiorly to the masseter muscle and traverse inferiorly along the mandibular ramus. The evaluation of masseter muscles was performed using pre-contrast T2-weighted MRI images without fat saturation, using a 20-channel head and neck coil from either the MAGNETOM Skyra 3T or MAGNETOM Avanto Fit 1.5T, manufactured by Siemens Healthcare in Erlangen, Germany. The parameters used for pre-contrast T2-weighted images without fat saturation were as follows: for the 3T MR service, the TR was 7060 ms, TE was 79 ms, slice thickness was 3 mm, matrix was 224 × 320, field of view (FOV) was 220 × 220 mm, number of excitations was 1, and flip angle was 180; for the 1.5T MR service, the TR was 5550 ms, TE was 102 ms, slice thickness was 3 mm, matrix was 217 × 320, FOV was 230 × 230 mm, number of excitations was 2, and flip angle was 150.

**Figure 1:**
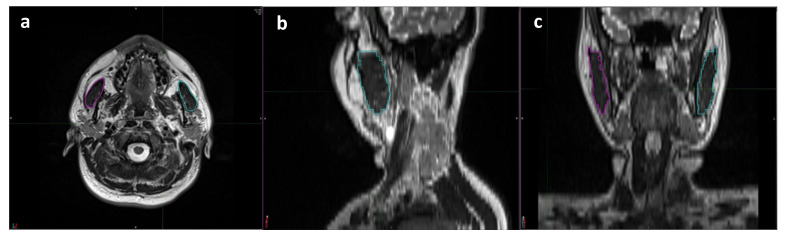
Representative delineation process used for volumetric measurements of each masseter muscle using T2 weighted magnetic resonance imaging scans: (**a**) axial, (**b**) sagittal, and (**c**) coronal planes (magenta: right masseter muscle; cyan: left masseter muscle).

The masseter muscle, which arises from the zygomatic arch and inserts into the mandibular angle, is the largest of all the muscles involved in mastication. MRI and planning CT scans obtained for SIB-IMRT planning were co-registered for the masseter muscle delineation and TMMV (total volume of right and left masseter muscles) calculation processes. A skilled radiologist (UAP) who specializes in head and neck imaging performed all radiologic evaluations, measurements, and analyses (Figure 1). The radiologist performed volumetric analyses on the treatment planning system while maintaining a blind approach to the clinical data. 

### 2.5. Baseline and Follow-Up Oral Evaluation and the Determination of RIT

An oral and maxillofacial surgeon with extensive experience (ES) conducted all oral assessments and MMO measurements. RIT was defined as having an MMO of 35 mm or less according to the standards set by Dijkstra et al. [20]. To measure MMO, we utilized Therabite^®^ from Atos Medical AB in Hörby, Sweden [21]. Patients were instructed to open their mouths as widely as possible while wearing the Therabite^®^ motion scale to measure the distance between the lower edge of one of the upper central incisors and the corresponding upper edge of one of the mandibular central incisors. The mean MMO was determined as the average numerical value of three consecutive measurements during each session. Measurements of MMO were repeated for each patient at 1, 3, 6, 9, and 12 months after C-CRT to evaluate the status of RIT. Subsequent measurements were carried out as required, or at every scheduled appointment thereafter.

### 2.6. Statistical Analysis

The primary intent of this retrospective research was to investigate the potential association between pre-TMMV readings and RIT rates. The last day of C-CRT was used as a reference point for determining the timing of the RIT diagnosis. The patients’ data were censored in cases where there was a diagnosis of RIT, a loss of follow-up, or death. The categorical data were displayed via percentage frequency distributions, while the continuous variables were shown utilizing median or mean values and ranges, as indicated. We conducted a receiver operating characteristic (ROC) curve analysis to determine the cutoff value(s) for continuous variables, which would allow us to divide the research cohort into two groups with substantially different outcomes. Logistic regression analysis was used to evaluate the individual significance of each variable in the multivariate analysis. All tests utilized a two-tailed *p*-value with a significance level of <0.05.

## 3. Results

The present retrospective cohort study examined the data of 112 patients diagnosed with LA-NPC who underwent C-CRT. As shown in Table 1, the median age was 60 years (range: 18–79), and 80 (71.4%) patients were male. Patients who had previously used tobacco products or alcohol made up 67.0% and 30.4%, respectively. The majority of patients had T3-4 (N = 65, 58.0%) or N2-3 (N = 75, 67.0%) advanced disease stages. The median pre-C-CRT MMO measurement was 41.6 mm (with a range of 37.0–46.8 mm). After the final post-C-CRT measurements, the median MMO decreased by 2.6 mm, which represents a decrease of 6.25% (Table 1 and Table 2). Based on Dijkstra’s widely accepted MMO ≤ 35 mm criterion, 28 patients were diagnosed with RIT, resulting in a prevalence rate of 25.0% [22]. The median time from the completion of C-CRT to the RIT diagnosis was 9 months (range: 4–18 months).

**Figure 2:**
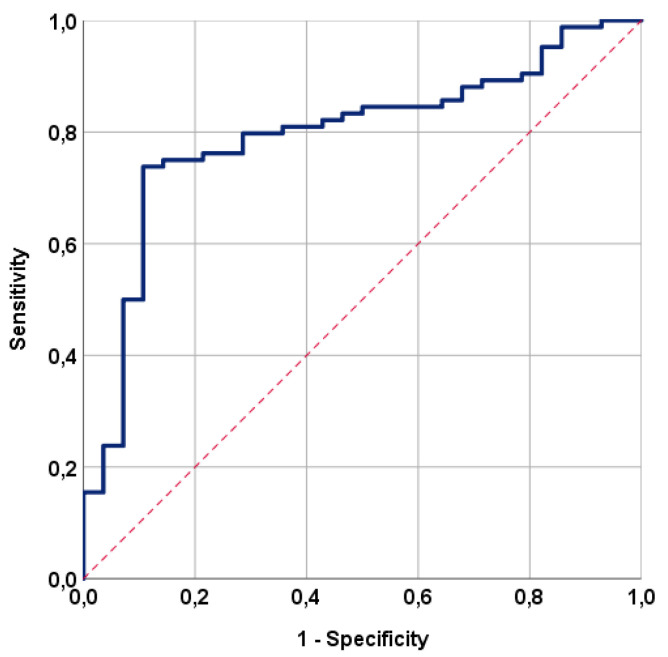
The outcomes of a receiver operating characteristic (ROC) curve analysis examining the correlation between the pre-treatment total masseter muscle volume and radiation-induced trismus rates following concurrent chemoradiotherapy [area under the curve (AUC): 79.5%; sensitivity: 75.0%; specificity: 78.6%; Youden index: 0.536].

Using ROC curve analysis, we sought a possible significant link between RIT incidence rates and pre-C-CRT TMMV measurements. The ideal TMMV cutoff was 35.0 cc [area under the curve (AUC): 79.5%; sensitivity: 75.0%; specificity: 78.6%; Youden index: 0.536] (Figure 2). As a result, the cutoff value was used to divide the entire research population into two groups: Group 1: TMMV ≤ 35.0 cc (N = 43) and Group 2: TMMV > 35.0 cc (N = 69). The two TMMV groups had similar distributions of baseline and treatment characteristics (Table 1 and Table 2). However, the incidence of RIT was significantly higher in patients with TMMV ≤ 35.0 cc compared to those with TMMV > 35.0 cc [51.2% vs. 8.7%; HR: 6.79; *p* < 0.001] (Table 3). Further analysis revealed that the mean C-CRT to RIT diagnosis interval was also significantly shorter in the TMMV ≤ 35.0 cc group compared to the TMMV > 35.0 cc group (9.6 months vs. 13.8 months; *p* = 0.02) (Table 2).

**Figure 3:**
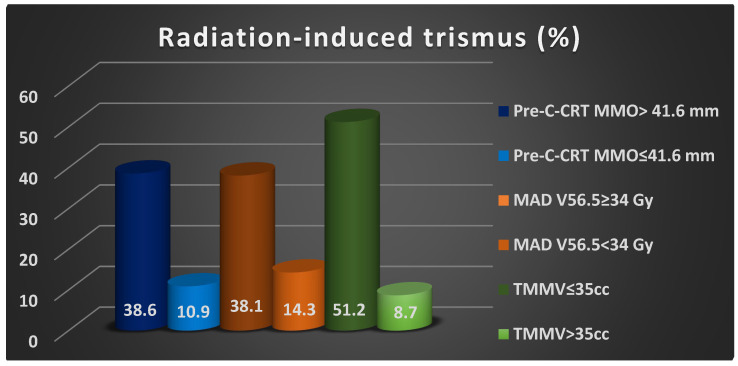
The bar chart depicting the rates of radiation-induced trismus rates per factor demonstrated independent significance in multivariate analyses. Abbreviations: C-CRT; concurrent chemoradiotherapy, MMO; maximum mouth opening, MAD; masticatory apparatus dose, V; volume, TMMV; total masseter muscle volume, cc; cubic centimeter.

Univariate analyses revealed a significant relationship between RIT incidences and pre-C-CRT groups of MMO (38.6% vs. 10.9% for >41.6 mm, *p* = 0.001), MAD V56.5 group (14.3% vs. 38.1% for ≥34% Gy, *p* = 0.002), and TMMV group (51.2% vs. 8.7% for >35 cc, *p* ˂ 0.001), with the former groups exhibiting higher RIT rates (Table 3). Multivariate logistic regression analyses confirmed that all three characteristics were independent and significant predictors of RIT in this patient group (*p* < 0.05 for each) (Table 3, Figure 3). 

## 4. Discussion

The main objective of this retrospective study was to determine the feasibility of utilizing pre-C-CRT TMMV levels as a predictor for the occurrence of RIT in patients with LA-NPC. Our most remarkable findings were the demonstration of a robust and independent association between pre-C-CRT TMMV 35.0 cc and a higher incidence of RIT [51.2% vs. 8.7% for TMMV > 35.0 cc; HR: 6.79; *p* < 0.001] and a significantly shorter mean C-CRT to RIT diagnosis interval (9.6 months vs. 13.8 months for TMMV > 35.0 cc; *p* = 0.02).

Pre-C-CRT MMO and MAD measures are two significant factors associated with RIT incidence rates following RT or C-CRT [22,23,24]. In this regard, our findings demonstrated a pre-C-CRT MMO ≤ 41.6 mm (38.6% vs. 10.9% for pre-C-CRT MMO > 41.6 mm, *p* = 0.001) and a MAD V56 > 34% (38.1% vs. 14.3% for MAD V56 ≤ 34%, *p* = 0.002), confirming narrower baseline MMO and higher MAD measurements as strong predictors of RIT in this patient group. Similar to our findings, Kraaijenga et al. [23], Owosho et al. [22], and Somay et al. [9] reported baseline MMO measurements of <46 mm, <40 mm, and ≤40.7 mm as significant predictors of increased RIT rates. While there is limited research available on MAD, studies have indicated that higher mean doses of RT administered to the components of the masticatory apparatus (measured as a dosimetric parameter for 100% volume) are linked to higher rates of radiation-induced toxicity such as RIT [9,11]. Based on the findings of the limited number of studies currently available and our research, it can be deduced that the mean and Vx (volume receiving X Gy) MAD values have the potential to function as indicators for assessing the extent of damage in the masticatory apparatus following RT, which is a functional parallel organ in terms of radiobiology. Therefore, every effort should be made to achieve as low a MAD value as feasible during the RT planning process to reduce the RIT rates.

The most notable discovery of our research was that patients presenting with a TMMV ≤ 35.0 cc had higher RIT incidence rates than those with a TMMV > 35.0 cc [51.2% vs. 8.7%; *p* < 0.001]. To our best knowledge, this finding is the first to establish a significant connection between RIT rates and TMMV in LA-NPC patients treated with definitive C-CRT. Previous research has linked radiation-induced inflammation to the difficult-to-predict RIT. It has been proposed that biomarkers derived from pre-treatment blood parameters could be used to predict RIT rates [9,11]. Previous research has not yet explored the value of pretreatment masseter muscle volume in predicting the rate of RIT. However, it is well recognized that masseter muscle abnormalities are established factors in the development of trismus, regardless of the cause, including RIT. In the first study of its kind, using the masseter muscle cross-sectional area (MMCSA) method, Wallace et al. [25] investigated the masseter muscle, which revealed a strong correlation between sarcopenia and MMCSA. Based on the findings of this research, McGoldrick et al. [26] assessed the usefulness of the same methodology in head and neck cancer patients. Their investigation found that sarcopenia, as indicated by MMCSA, may have prognostic significance in senior patients (HR: 1.74, *p* = 0.042). However, regardless of the methodological differences, these collective findings consistently highlight the dependability of masseter muscle measures as a novel biomarker for predicting treatment-related toxicities and survival outcomes.

Muscle wasting in cancer patients is a poor prognostic factor frequently associated with pre-cachexia or cachexia [27]. This condition is characterized by the unintentional loss of muscle mass in the absence of surgical or other physical damage to the muscle fibers, nerves, and/or blood vessels. The loss of skeletal muscle mass experienced during the development of cancer cachexia is mainly caused by a combination of protein degradation and reduced protein synthesis. This process is triggered by the increased production of inflammatory mediators and the activation of specific transcription factors and signaling pathways [28,29]. Given these facts, the significant association between TMMV and RIT rates in our patient cohort manifesting with a pre-C-CRT TMMMV < 35 cc may imply a condition of cancer cachexia in progress and related weakened muscle mass and strength-inducing trismus in this particular patient cohort. This implication is indirectly supported by a recently reported study by Pehlivan et al., where the authors discovered significant associations between lower pre-treatment TMMV values and worse survival outcomes in LA-NPC patients treated with definitive C-CRT, as a pre-cachectic condition may have impacted the results [16]. Another logical explanation could be the collaboration of two hyperinflammatory states, namely cancer cachexia and radiation-induced tissue injury, which resulted in increased inflammatory mediator secretion, increased proteolysis, facilitated sarcopenia and fibrosis in the masseter muscles, and ultimately RIT development [30,31].

Another key finding in our study was the demonstration of a significant correlation between a smaller TMMV and a shorter mean duration between the completion of C-CRT and RIT manifestation (9.6 months vs. 13.8 months for TMMV ≤ 35.0 cc; *p* = 0.02). While the precise explanation requires more investigation, this discovery might potentially be attributed to the possibility that a reduced TMMV may serve as an early indicator of a currently underway insult affecting the muscle mass, such as cancer cachexia. Furthermore, it is possible that the impact of radiation-induced damage on muscle fibers, vasculature, and nerves was more significant in the smaller volume and, conceivably, the weaker masseter muscles, which might have led to an earlier onset of muscle fibrosis and resultant RIT in these individuals [32]. Regardless of the precise underlying causes, based on our current knowledge, the present finding indicates that most instances of RIT will occur within a time frame of less than one year in individuals presenting with smaller baseline TMMV measures. Despite the need for confirmatory research results, we contend that providing nutritional assistance and implementing preventative measures for RIT at an earlier stage might be a prudent strategy to reduce the incidences of cancer cachexia and RIT in such patients. 

Our study possesses several strengths: first, the study consisted of a select group of LA-NPC patients treated with the same concurrent C-CRT and medication regimen. Second, while CT is commonly used in RT planning, incorporating co-registered CT-MRI scans may have enabled a more precise determination of TMMV by facilitating the identification of exact muscle borders. However, our study has some drawbacks as well: first, it has a retrospective design with a small cohort size, which may introduce unintentional selection bias-related issues. Second, as a consequence of the limited number of participants in our study, we were unable to validate the findings by using either external or internal control groups. Third, no probable correlation was examined between the pretreatment TMMV and biomarkers of cancer cachexia, such as cytokines and acute phase reactants. Fourth, because our study was limited to LA-NPC patients, it becomes arduous to draw inferences regarding TMMV’s ability to predict RIT and other toxicities in other head and neck cancers. We believe that a decrease in the volume of the masseter muscles, which are the strongest muscles used for chewing, may indicate the patient’s poor nutritional status and/or a pre-cachectic status with a potential to progress towards irreversibly fatal cancer cachexia. Despite being sensible, we believe that well-designed research on this vital subject is required to substantiate our opinion. And fifth, due to the lack of dynamic measurement of masseter muscle volume during and after treatment in the current study, the optimal threshold value could not be established. Hence, future research should use dynamic measurements to establish the best cutoff value, which may better stratify patients according to RIT and, presumably, other toxicity risks. Given these facts, we may have missed the chance to discover the exact processes that connect the first TMMV measurements with RIT rates. Undoubtedly, conducting additional well-designed and large-scale research is essential to address the issues associated with RIT in patients at a higher risk after RT or C-CRT. Such research outcomes can help initiate individualized nutritional support and timely preventive measures, which can play a pivotal role in mitigating the incapacitating effects of RIT.

## 5. Conclusions

Our research findings indicate that pre-treatment TMMV values can be used as an innovative biological predictor of RIT rates in LA-NPC patients who undergo definitive C-CRT treatment. If additional studies confirm these findings, we may be able to categorize these patients based on their risk of RIT and develop follow-up protocols tailored to their specific risk levels. 

## Figures and Tables

**Table 1:** Baseline and treatment characteristics for all patients and per total masseter muscle volume groups.

Characteristics	All Patients (N = 112)	TMMV ≤ 35 cc (N = 43)	TMMV > 35 cc (N = 69)	*p*-Value
Median age, y (range)	60 (18–79)	59 (28–73)	61 (18–79)	0.82
Age group, n (%)				0.56
≥60 years	57 (50.9)	20 (46.5)	37 (53.6)	
<60 years	55 (49.1)	23 (53.5)	32 (46.4)	
Gender, N (%)				0.29
Female	32 (28.6)	28 (65.1)	17 (24.6)	
Male	80 (71.4)	15 (34.9)	52 (75.4)	
Smoking status, N (%)				0.06
No	37 (33.0)	19 (44.2)	18 (26.1)	
Yes	75 (67.0)	24 (55.8)	51 (73.9)	
Alcohol consumption, N (%)				0.83
No	78 (69.6)	29 (67.4)	49 (71.0)	
Yes	34 (30.4)	14 (32.6)	20 (29.0)	
Median pre-C-CRT MMO, mm (range)	41.6 (37.0–46.8)	41.0 (47.8–45)	41.9 (38.9–46.8)	0.027 *
Pre-C-CRT MMO group, N (%)				0.05 *
≤41.6 mm	57 (50.9)	27 (62.8)	30 (43.5)	
>41.6 mm	55 (49.1)	16 (37.2)	39 (56.5)	
T stage, N (%)				0.70
1–2	47 (42.0)	17 (39.5)	30 (43.5)	
3–4	65 (58.0)	26 (60.5)	39 (56.5)	
N stage, N (%)				0.30
0–1	37 (33.0)	17 (39.5)	20 (29.0)	
2–3	75 (67.0)	26 (60.5)	49 (71.0)	

Abbreviations: TMMV; total masseter muscle volume, C-CRT; concurrent chemoradiotherapy, MMO; maximum mouth opening, T; tumor, N; node. * *p* < 0.05

**Table 2:** Treatment characteristics and outcomes per total masseter muscle volume groups.

Characteristic	All Patients (N = 112)	TMMV ≤ 35 cc (N = 43)	TMMV > 35 cc (N = 69)	*p*-Value
Concurrent chemotherapy cycles, N (%)				0.76
1	20 (17.6)	8 (18.6)	12 (17.4)	
2–3	92 (82.4)	35 (81.4)	57 (82.6)	
Adjuvant chemotherapy cycles, N (%)				0.32
0	29 (25.9)	13 (30.2)	16 (23.4)	
1–2	83 (74.1)	30 (69.8)	53 (76.6)	
Mean MAD, Gy (range)	35.2 (11.9–66.8)	34.9 (11.9–65.7)	35.6 (12.3–66.8)	0.87
MAD V56.5, N (%)				0.54
<34 Gy	70 (62.5)	28 (65.1)	42 (60.7)	
≥34 Gy	42 (37.5)	15 (34.9)	27 (39.3)	
Mean C-CRT to RIT interval, mo. (range)	9.5 (4–18)	9.6 (4–18)	13.8 (9–18)	0.02 *
Median post-C-CRT MMO, mm (range)	39.0 (25.0–42.0)	34.0 (25.9–41.0)	39.2 (30.7–42.0)	0.05
RIT, N (%)				˂0.001
Present	28 (25.0)	22 (51.2)	6 (8.7)	
Absent	84 (75.0)	21 (48.8)	63 (91.3)	

Abbreviations: TMMV; total masseter muscle volume, cc; cubic centimeter, MAD; masticatory apparatus dose, V; volume, C-CRT; concurrent chemoradiotherapy, MMO; maximum mouth opening, RIT; radiation-induced trismus. Represents * *p* < 0.05.

**Table 3:** Results of univariate and multivariate analysis.

Factors	RIT (%)	Univariate *p*-Value	Multivariate *p*-Value	HR (95% CI)
Age group (≥60 years vs. <60 years)	19.3 vs. 30.9	0.19	-	1.54 (0.94–2.07)
Gender (male vs. female)	22.5 vs. 31.3	0.34	-	1.28 (0.87–163)
Smoking status (no vs. yes)	22.7 vs. 29.7	0.50	-	1.14 (0.96–1.33)
Alcohol consumption (no vs. yes)	23.1 vs. 29.4	0.50	-	1.09 (0.78–1.76)
Pre-C-CRT MMO group (≤41.6 mm vs. >41.6 mm)	38.6 vs. 10.9	0.001	0.007	3.71 (2.68–5.19)
T-stage group (1–2 vs. 3–4)	23.4 vs. 26.2	0.83	-	1.04 (0.88–1.23)
N-stage group (0–1 vs. 2–3)	24.3 vs. 25.3	1.00	-	1.02 (0.95–1.07)
Concurrent chemotherapy cycles (1 vs. 2–3)	23.3 vs. 26.1	0.87	-	1.12 (0.89–1.24)
Adjuvant chemotherapy cycles (0 vs. 1–2)	24.1 vs. 25.3	0.61	-	1.07 (0.94–1.11)
MAD V56.5 group (<34 Gy vs. ≥34 Gy)	14.3 vs. 38.1	0.002	0.004	2.58 (1.74–3.64)
TMMV group (≤35 cc vs. >35 cc)	51.2 vs. 8.7	˂0.001	<0.001	6.79 (4.87–9.16)

Abbreviations: RIT; radiation-induced trismus, HR; hazard ratio, C-CRT; concurrent chemoradiotherapy, MMO; maximum mouth opening, T; tumor, N; node, MAD; masticatory apparatus dose, V; volume, TMMV; total masseter muscle volume, cc; cubic centimeter.

## Data Availability

The present data belong to and are stored at the Baskent University Faculty of Medicine and cannot be shared without permission. For researchers who meet the following criteria for access to confidential data, contact the Baskent University Corporate Data Access/Ethics Board: adanabaskent@baskent.edu.tr.
